# Squat Detection of Railway Switches and Crossings Using Point Machine Vibration Measurements

**DOI:** 10.3390/s23073666

**Published:** 2023-03-31

**Authors:** Yang Zuo, Jan Lundberg, Taoufik Najeh, Matti Rantatalo, Johan Odelius

**Affiliations:** Operation and Maintenance Engineering, Luleå University of Technology, 97187 Luleå, Sweden

**Keywords:** railway switch & crossing, vibration, squats, condition monitoring, wavelet denoising, fault detection

## Abstract

Railway switches and crossings (S&C) are among the most important high-value components in a railway network and a failure of such an asset could result in severe network disturbance. Therefore, potential defects need to be detected at an early stage to prevent traffic-disturbing downtime or even severe accidents. A squat is a common defect of S&Cs that has to be monitored and repaired to reduce such risks. In this study, a testbed including a full-scale S&C and a bogie wagon was developed. Vibrations were measured for different squat sizes by an accelerometer mounted at the point machine. A method of processing the vibration data and the speed data is proposed to investigate the possibility of detecting and quantifying the severity of a squat. One key technology used is wavelet denoising. The study shows that it is possible to monitor the development of the squat size on the rail up to around 13 m from the point machine. The relationships between the normalised peak-to-peak amplitude of the vibration signal and the squat depth were also estimated.

## 1. Introduction

Railway switches and crossings (S&C) play an important role in a railway system by enabling trains to switch between different tracks. To achieve such functionality, S&C include movable components. This, together with the discontinuities in the rail geometry and variability in the track support stiffness, cause higher failure rates compared with plain line tracks [1].

Therefore, the maintenance cost of S&C often comprises a considerable part of the total maintenance budget of a railway system. In Sweden, approximately 8 percent of the budget was dedicated to S&C maintenance in 2009 [2]. In 2018, S&C maintenance consumed SEK 530 million, which is almost 10 percent of the total maintenance cost [3]. It is reported that S&Cs in the United Kingdom could consume up to one-third of the whole maintenance budget [4]. The cost varies from country to country, depending on S&C type and deployed maintenance strategy. However, the maintenance cost is not the only cost that should be taken into account. A more complete calculation of Life Cycle Cost (LCC) of S&Cs is analysed by Nissen A [5].

To avoid failures of these important junction points and to reduce the cost, condition monitoring of S&Cs and shifting from corrective maintenance to preventive maintenance are needed. Many researchers have proposed different condition monitoring technologies to detect defects that could lead to failures of S&Cs. One study monitors the motor current and the force in the drive bar to detect switch defects using the Kalman filter [6]. Another study utilises qualitative trend analysis of current, force and displacement to detect the faults [7]. Previous studies focused on measuring different parameters of S&Cs such as the voltage, current, force, displacement, etc., of the point machine during the switching procedure. Those data are later utilised to detect anomalies associated with possible failures or degraded statuses. A review of the existing fault detection diagnostics (FDD) techniques for railway S&Cs was performed by Moussa, A. [8]. Different anomaly detection techniques are used to process the data and detect possible defects, such as using a rule-based decision process with the help of the Kalman filter [9] and net energy analysis (NEA) [10]. Such measurement data can also be applied to implement a closed-loop feedback control of a lift-and-drop railway track switch actuator [11]. These approaches all focus on defects of the functionality of a point machine. However, other parts of S&Cs also need to be monitored and assessed, such as different rail parts including the stock rail, the switch rail and the crossing nose/frog.

Rail surface defects can be detected by using manual on-site inspections, vehicle-based measurements using visual systems (camera/laser) [12,13] or eddy current systems [14,15]. A manual inspection could be subjected to the effect of human errors and can expose inspectors to hazardous situations. The method of using a dedicated track inspection vehicle/eddy current system would acquire accurate measurements, but the frequency of measurements would be limited due to the high cost and long track possession time. In the past decade, with the price of sensors decreasing, there are more studies that use inertial sensors mounted on the train to detect faults on the tracks. Lederman, G. introduced an implicit model and an explicit model for track monitoring with vibration data from in-service trains [16]. Liu, J. et al. proposed a variational autoencoder (VAE) approach to identify the anomalies of longitude track geometry [17]. Bridgelall, R. et al. presented a method that combines multiple signals from each traversal to increase the signal quality for identifying track anomalies [18]. There is also a study that reviews the current techniques used for track geometry condition monitoring by utilising data from in-service vehicles [19]. This approach usually collects vibration data from sensors mounted on the bogie/axle box to identify rail defects. The disadvantage of such an approach is that the collected vibration signals are dependent on the type of axles and the wear condition on the wheel axle bearings. Furthermore, it is designed to inspect a whole track line instead of focusing on monitoring the defects of an S&C. When it comes to a dedicated condition monitoring system for monitoring the rail conditions of S&Cs, there is very little work that has been performed. The purpose of this study is to reduce the cost of S&C maintenance, increase the reliability and reduce severe S&C failure such as switch blade breakage that may lead to catastrophic accidents. This study focuses on investigating the possibility to utilise the protective environment provided by the point machine and the available power supply to install accelerometers to detect squat defects.

There are many defects and failure modes of S&Cs. A squat is one common defect of the S&C. A study conducted by Ilaria, G. et al. stated that the most common cause of failure at the crossing panel is a squat on a casting, which comprises approximately one-third of all S&C failures [20]. The development and cause of squats are discussed and explained in detail in a study presented by Grassie, S.L. [21]. Squats at an early stage could be difficult to identify. There has been some previous research dedicated to modelling the process of squats’ development. Daniel W.J. pointed out that the rate of squat growth measured corresponds to a power law with a low exponent [22]. Freimanis A. highlighted the approach of peridynamic modelling of squat distribution and growth [23]. However, these types of models were developed to predict the growth of squats but not to detect them. Another study uses a parametric approach to analyse the axle box acceleration (ABA) signal and visualised the relationship between the signature tone and the train speed. It also states that high-frequency components (e.g., 1000 and 2000 Hz) could occur for light squats [24]. Another study presented a method for squat detection using wavelet analysis of vibration data extracted from ABA [25]. It introduced a new system for automatic squat detection. However, with this approach, a large amount of repetition data are needed to cancel out the random noise. Furthermore, this method is applied generally to all tracks and is not specifically focused on identifying squats at S&Cs. Another study focused on explaining the relationship between white etching layers (WELs) and the forming of squats; it proposes using more regular and relatively shallow grinding to control the development of the squats [26]. However, such an approach could potentially increase the maintenance frequency, and it has not been proven that the total costs would be lower. Zuo, Y. et al. presented a method of applying an isolation forest algorithm to monitor the overall status of an S&C related to squat defects [27]. However, the locations and severities of individual squats were not investigated.

While most of the previous studies were performed using ABA or BA to estimate squats on normal rails, this study proposed a new approach of investigating the possibility of detecting and quantifying squats of different severities by processing the vibration signal measured at the point machine. This embedded approach has the advantage of providing the accelerometers with a protective environment against rough weather conditions. It also offers available power supplies for the measuring equipment. The relationship between the vibration amplitude and the severity is estimated. By detecting the squats and following the evolution of the squat severity, maintenance actions could be planned and scheduled more efficiently.

## 2. Materials and Methods

### 2.1. Track Layout and the Testbed

**Figure 1:**
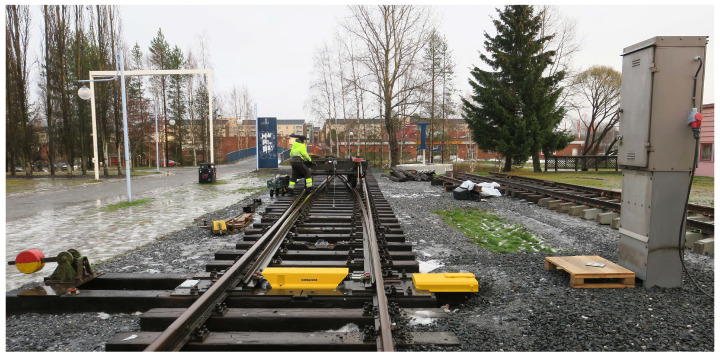
Test site. Adapted from Zuo, Y. et al. [27].

In this study, a method is presented to investigate the possibility of detecting and quantifying squat defects on an S&C. The experiment was performed in a testbed including a full-scale S&C and a bogie wagon. The accelerometer was mounted on the point machine. This setup provides a robust and protective environment for the accelerometer with easy access to electrical power. The vibration signal and the corresponding speed information were measured and saved while the bogie was travelling along the S&C with artificially introduced squats. The main layout of the testbed is shown in Figure 1. Another related study was performed with the same S&C to estimate the wear size using deep learning [28].

**Figure 2:**
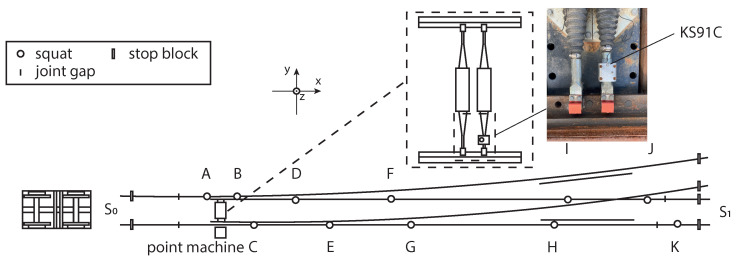
Test setup schematic diagram and accelerometer placement. Adapted from Zuo, Y. et al. [27].

**Figure 3:**
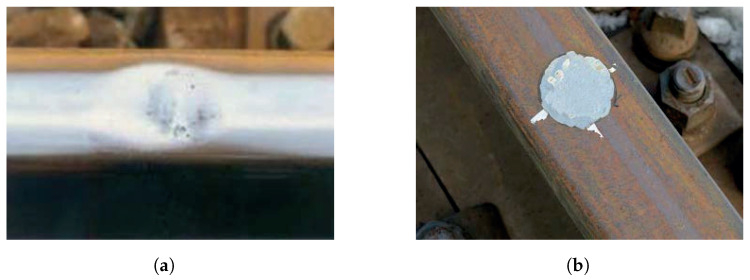
A real and an artificial squat: (**a**) A real squat. (**b**) An artificial squat in the testbed.

The S&C used in the experiment has a dimension of 1:16 and a length of 38.14 m; it is located at Luleå University campus. A bogie was used to travel through the S&C to generate the vibration. A simplified illustration of the testbed is shown in Figure 2. The rails are labelled in the figure from rail 1 to rail 4. The squats are labelled from A to K. To simulate two different squat levels, the squats were manually introduced step-wisely with 1 mm and 4 mm maximum depth. The actual positions and the measured dimensions of the squats are listed in Table 1. The squat with 1 mm depth is around 42 mm in diameter and the squat with 4 mm depth is about 63 mm in diameter. $S_{0}$ and $S_{1}$ are two stop blocks of the S&C on each end in the through direction. Wheels 2 and 4 travel first on rail 2 and then switch to rail 3 while wheels 1 and 3 always travel on rail 1. The point machine is located 5.86 m away from stop block $S_{0}$. Figure 3 shows what a real squat and an artificially introduced squat in the testbed look like. The artificial squats were introduced by a conventional angle grinder.

### 2.2. Measurement Setup

#### 2.2.1. Accelerometer

A previous study shows that the expected movements of the trains hardly generate frequencies higher than 20 kHz [29]. Molodova, M. et al. [24] further stated that squat-related frequencies in their system have high energy peaks around 300 Hz and 1060–1160 Hz. Furthermore, the high-frequency response component could reach up to 2 kHz. Some pre-tests were performed with the testbed, and it showed that most of the impact responses of interest could be captured within the 10 kHz range. However, to be able to catch possible higher frequencies, an accelerometer that can measure from 0.3 to 37,000 Hz was installed. Some key technical parameters of the accelerometer used are listed in Table 2.

The accelerometer used in this study was mounted inside the point machine on the rods that were connected to the switch blade. The detailed installation location of the accelerometer can be seen in Figure 2. Here x, y and z coordinates are defined. The mounted accelerometer was used to measure the vibration in the z-direction. On the upper-right corner is a photo taken from the test site to show the sensor used and its positioning.

#### 2.2.2. Data Acquisition

The point machine used in the experiment is electrical mechanical. During the experiment, the vibration signal and the corresponding speed information were measured and logged when the bogie was moving along the rail of the S&C from one end to the other.

A system was built to measure and save the vibration and speed data. The accelerometer measured the acceleration, which was then acquired by a conventional data acquisition system (DAQ). An optical sensor was utilised to estimate the wheel speed due to the bogie being manually pushed and the moving speed being non-constant. Both data were later acquired by Arduino Uno Wi-Fi V2. The system was implemented in VI code running in LabVIEW 2019 and saved onto an external hard drive. The sampling frequency of the system is 51.2 kHz.

### 2.3. Test Runs

The experiment was performed as follows. Squats were introduced step-wise with 2 squat levels at 1 mm and 4 mm depths. Tests were also performed before the squats were created as base line references. The test runs and the recorded data were organised as shown in Table 3.

### 2.4. Post-Processing

**Figure 4:**
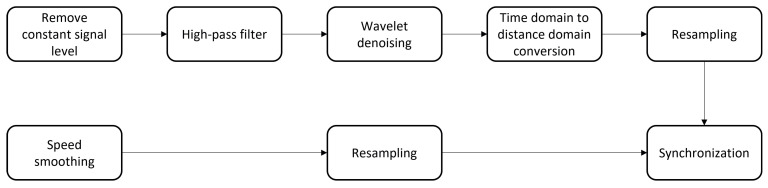
Post-processing procedure.

The post-processing procedure of the measured signals is described in Figure 4. The vibration signal was high-pass filtered, wavelet denoised and then synchronised with the smoothed and re-sampled speed data. After synchronising the vibration and speed data, the vibration data were converted from the time domain to the spatial domain. Then the data were re-sampled to the same sampling frequency as the original time domain signal. The expected events chart was used to match the final processed vibration signal and identify impact events. The steps are described in detail in the following paragraphs.

#### 2.4.1. High-Pass filtering

Since the focus of this study was to detect impact events, a third-order high-pass filter with a cut-off frequency at 100 Hz was applied to the original signal to remove low-frequency components such as the variant mean value.

#### 2.4.2. Wavelet

To emphasise the impact events in the acceleration signal and to reduce noise, wavelet denoising was performed. Wavelets have been applied as a denoising technology for vibration data [30,31] ever since it was first introduced by Donoho and Johnstone [32]. The wavelet transform is a very powerful tool for time–frequency analysis and the cornerstone of wavelet denoising. It can be viewed as replacing the short-time Fourier transform’s “time-frequency window” $g_{t,\xi }$ with a “time-scale window” $\Psi_{a,b}$. The continuous wavelet transform can be defined as follows.

A function Ψ with

(1) $$\int_{R}^{}\Psi \left(x\right)dx=0$$

is called a wavelet. For every f, Ψ defines the continuous wavelet transform

(2) $$W_{\Psi }f\left(a,b\right)=\int_{R}^{}f\left(x\right)\bar{\Psi (\frac{x-b}{a})}dx\;\mathrm{for}\;\mathrm{all}\;a,b\;\in R_{+}\times R$$

where

(3) $$\Psi_{a,b}=\frac{1}{\sqrt{a}}\Psi \left(\frac{x-b}{a}\right)$$

Here *a* is called the scale factor and represents the scaling of the function, and *b* is called the shift factor and represents the temporal offset of the function. The function Ψ is called the mother wavelet. It is chosen to be localised at x = 0 and at some $\omega =\omega_{0}>0$ (and/or $\omega =-\omega_{0})$.

The wavelet denoising technology utilises both wavelet transform and reverse wavelet transform. First, a wavelet transform was conducted and the values of the coefficients underneath a certain threshold were considered as noise and were thereby scaled down or set to zero. Then a reverse wavelet transform was applied to reconstruct a denoised version of the original signal. More details of the wavelet denoising technology implementation and application are presented in “Wavelet Denoising” by Luo and Zhang [33].

The implementation of wavelet denoising is as follows. The mother wavelet function used was sym4, and a level 9 decomposition was chosen since this setup showed good performance from a previous study [27]. The scaling factor was 0.65. The empirical Bayes denoising method was used and the posterior median threshold rule was used to measure the risk. “LevelDependent” was used to estimate the variance of the noise based on the wavelet coefficients at each resolution level. The running time for MATLAB with an Intel Core i7-8650U CPU and 16 G memory was around 13.1 s.

**Figure 5:**
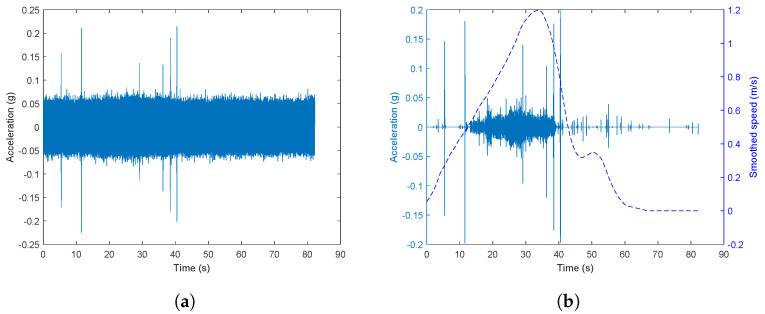
Comparison of the signal before and after wavelet denoising in no squat case. (**a**) The signal after applying the high-pass filter before denoising. (**b**) The signal after denoising.

The effect of wavelet denoising for the experiment data can be seen in Figure 5. It shows the acceleration signal in the time domain of one run from $S_{0}$ to $S_{1}$ with no manually introduced squats. Figure 5a shows the acceleration signal after applying the high-pass filter before wavelet denoising. Figure 5b shows the acceleration signal after after wavelet denoising. The dashed line represents the speed data after smoothing. In this case, the peaks seen in the acceleration signal are generated by different impact events during the experiments, such as when the wheels interact with the joints, for example. For the original signal, only a few impulse responses with a distinct amplitude were visible. However, the noise level was suppressed in the denoised signal, which enhanced the signal to noise ratio and more impact events become visible.

#### 2.4.3. Time to Spatial Domain Conversion

The original vibration data were sampled in the time domain. However, what is interesting in the study is where the squats are located. With the help of the logged speed information, it is possible to convert the time domain data into the spatial domain. Since the speed is not constant, the converted spatial domain signal will have a non-constant distance interval.

#### 2.4.4. Smoothing Speed Signal

Since in this experiment the bogie was pushed manually, the speed variation should be limited and there should not be any sudden change of speed. However, certain parts of the measured speed data had sudden changes due to measurement error. To remove such effects, convolution technology was used for smoothing the speed data before applying upsampling.

#### 2.4.5. Re-Sampling

Re-sampling was applied to both the speed signal and the vibration signal. Since the speed signal was sampled at 1 Hz and the vibration signal at 51.2 kHz, upsampling was applied to the speed signal. The converted signal in the spatial domain had a non-constant sampling frequency because the speed was not constant. Therefore, re-sampling to 51.2 kHz was applied using interpolation technology.

#### 2.4.6. Synchronising

To be able to perform the spatial domain conversion, the synchronising of the vibration signal and the speed estimation data is necessary. The actual starting time of the vibration signal and the speed signal was not exactly the same due to them being controlled by two different paths with the acceleration data transmitted via a cable and the speed data transmitted via WiFi. The signal was aligned by using the fact that both signals stop at the same time. Further, the signals where the measuring speed was constant zero were removed because that part of the signal was recorded before the bogie started to move and do not contribute to the analysis. The accuracy of the speed measurement could be one reason that the synchronisation was not perfect.

**Figure 6:**
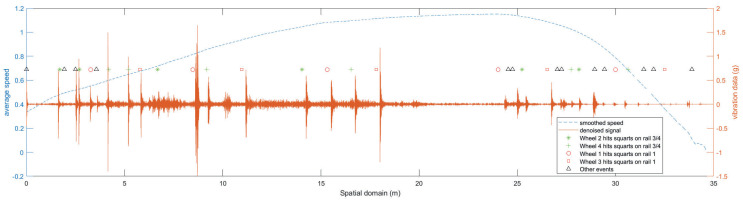
An example of 4 mm squat case aligned signal with all expected events. (Markers show where a wheel is expected to hit squats or other discontinuities along the rail).

The technique used to help synchronise the vibration signals in the spatial domain with the expected events is utilising signatory common impulse responses as reference points. The event chosen is when the front wheel hits the first rail joint, which happens in all test cases. This point is used to align the vibration signal in the spatial domain to the expected events. With such an approach, the acceleration data are better synchronised with the expected events. Figure 6 shows an example of the acceleration signal after post-processing of pushing the bogie from $S_{0}$ to $S_{1}$ with 11 squats with 4 mm depth. The corresponding speed curve is also presented with a dashed line. With the help of the generated expected events chart, it is apparent that after the matching, the expected events match the acceleration signal impulse responses. This is especially true for the first 7 squats. It is worth pointing out that because of using such a technique, the first impulse response will always be the event of front wheels hitting the first joint gap, which is shown at the distance 0 m.

#### 2.4.7. Expected Events Chart

In a railway system, the vibrations are generated by the wheel and rail interaction. The condition of the wheel and the rail will directly influence how these vibration signals look. Defects of the wheel or rail will generate larger impulse responses compared to a new system due to the increased dynamic force between the wheel and the rail surface. In the testbed, when a wheel hits squats, joint gaps or stop blocks, the amplitude of the vibration signal should increase. These increased impulse responses are called impact events in this paper. A chart of expected impact events was generated by utilising the physical location information of the squats, the joint gaps or the stop blocks. The expected events when the bogie travels from $S_{0}$ to $S_{1}$ are illustrated in Figure 6. Markers show different expected events. The star symbol represents when wheel 2 hits a squat, the plus symbol represents when wheel 4 hits a squat, the circle represents when wheel 1 hits a squat, the square represents when wheel 3 hits a squat and the triangle represents other events.

The signal was further sliced into segments to analyse the vibration signal near different events such as hitting a gap or encountering a squat. There are many different methods to define the amplitude of a signal. In this study, a maximum peak-to-peak amplitude is used.

## 3. Results and Discussions

### 3.1. Squat Detection

A 0.5 m window on both sides of the expected events chart was utilised to extract the corresponding impulse responses for different squats. Because of the limitation in speed measurement accuracy, not all squats could be detected. The results are listed in Table 4 for the 1 mm case and Table 5 for the 4 mm case, respectively. The results show that it is possible to extract and locate 4 out of 6 squats with a 1 mm depth that is within a 13.46 m range from the accelerometers. It is also possible to detect and locate 6 out of 7 squats with 4 mm depth within the same range. Squats located more than 22.16 m away can not be extracted and located correctly with the current implementation for both the 1 mm and 4 mm case. One possible reason is the error of speed measurement accumulates with time; thus, the further the squat is located from the starting point, the more uncertainty it has. Another reason is that the amplitude is smaller when the squat is further away from the accelerometer. The results show that it is possible to implement an automatic squat detection system with the processed vibration signal in the spatial domain within a certain distance.

### 3.2. General Trends of the Impact Events

**Figure 7:**
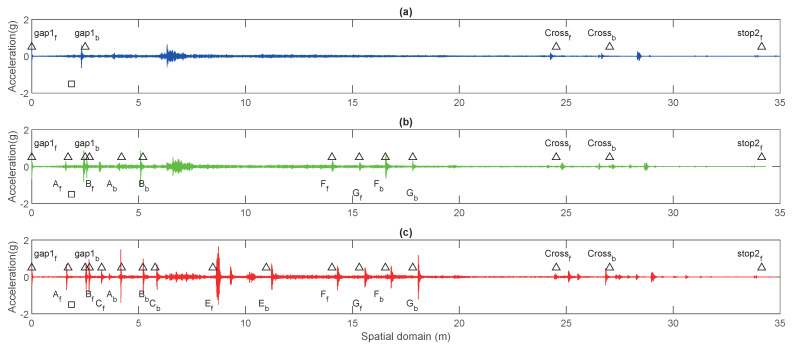
Amplitude comparison for different squat levels: (**a**) No squat case. (**b**) 1 mm depth of squats. (**c**) 4 mm depth of squats.

Figure 7 visualises the changing of impulse responses when the squat size increases. The first impulse response at 0 m is the event of the front wheel hitting the first joint gap. The first plot shows the reference case with no squats. The second plot is of 1 mm squat depth level case and the third one is 4 mm. Both the amplitude of the impulse response and the number of identifiable impulse responses increase with a larger squat depth. The amplitude decreases and the synchronisation becomes less accurate with increased travelling distance due to the accumulated speed measurement error. The square represents the location of the point machine. The triangles represent events of rails hitting squats, gaps, crossing nose and stops. The subscripts f and b represent if it is the front wheel or the back wheel, respectively, that is involved in the event. In general, the selected events match the acceleration signal impulse responses. This is true especially for the first seven squats. As a trend, the events further away from the measuring point have smaller impulse response amplitude.

Notably, although the switch is in the “through” status, and rail 1 is not directly attached to the point machine, the amplitudes of these events were still large. Some of them are even larger than the impulse response on rail 2 or 3, as can be seen in Figure 7. For example are squats G and F. The interpretation is that the vibration is transmitted through the bogie. How the wheels are positioned on the track when hitting the squat could be an important factor. However, to be able to draw a concrete conclusion, more data must be collected and analysed.

### 3.3. Normalising Peak-to-Peak Amplitude to the 1 m/s Case

**Figure 8:**
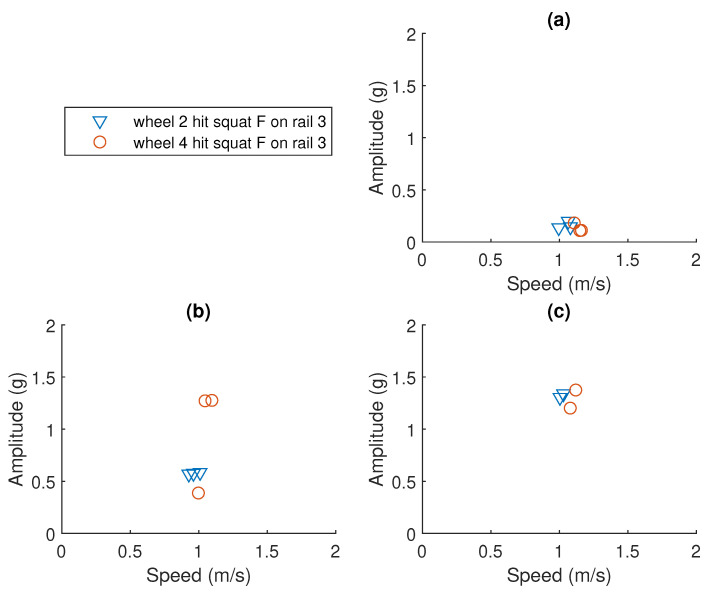
Peak-to-peak amplitude data for squat F on rail 3. (**a**) No squat case. (**b**) 1 mm squat case. (**c**) 4 mm squat case.

**Figure 9:**
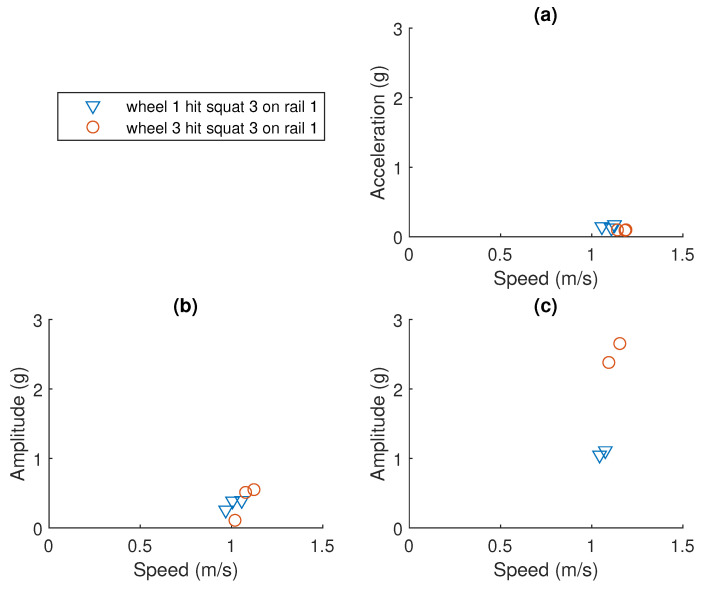
Peak-to-peak amplitude data for squat G on rail 1. (**a**) No squat case. (**b**) 1 mm squat case. (**c**) 4 mm squat case.

The maximum peak-to-peak amplitude data were extracted from the processed vibration signal with the help of the expected events chart. The original data of squat F and G are visualised in Figure 8 and Figure 9, respectively. As the speed of the bogie is not constant, the peak-to-peak amplitude values should not be directly compared. To estimate the relationship between the amplitude and the speed for each squat requires a large amount of repetition data at different speeds. In this study, such data are not available. From all the squats introduced, squat F and squat G were chosen to be further analysed. They have a few advantages that are listed below:

- They are located in the middle of the S&C and are not far from the accelerometer.
- They are not too close to the starting point where many events were happening at the same time.
- They are not too far away from the starting point either, so the amplitude value can be detected.
- They are close to each other and the speed of the bogie was within 1 ± 0.2 m/s.

For squat F and squat G, it is reasonable to assume a proportional relationship between the amplitude and the speed within such a small interval. All measured amplitudes for squat F and G are, therefore, divided by the corresponding speed to get an estimation of the amplitude at the speed of 1 m/s.

### 3.4. Statistics of the Estimated Amplitude for 1 m/s Case

**Figure 10:**
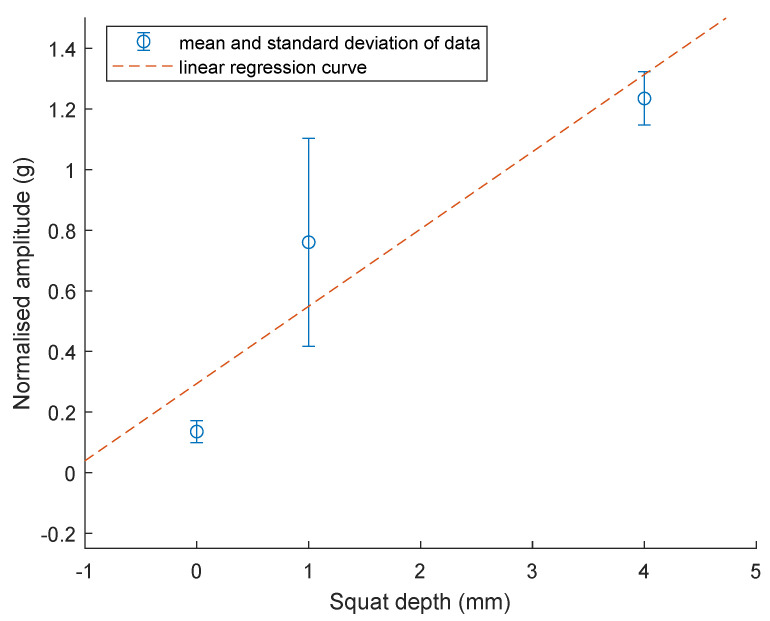
Mean and standard deviation of speed normalised amplitude vs. the squat depth. The dashed line is the fitted linear model. (wheel 2, 4 hit squat F on rail 3).

**Figure 11:**
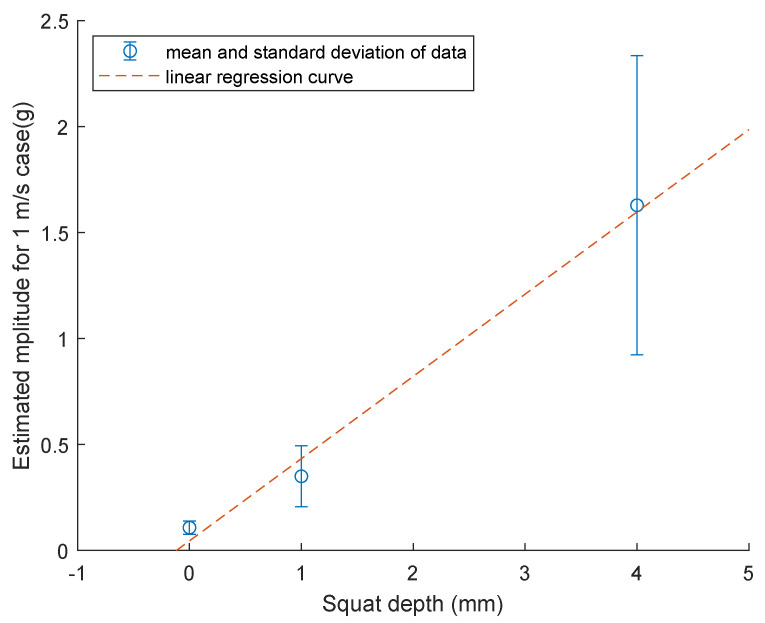
Mean and standard deviation of speed normalised amplitude vs. the squat depth. The dashed line is the fitted linear model. (wheel 1, 3 hit squat G on rail 1).

The mean and the standard deviation of the estimated acceleration is plotted against the corresponding squat depths of no squat, 1 mm squat and 4 mm squat for squat F. The results are visualised in Figure 10. It shows that the mean amplitude value increases from 0.7605 g to 1.2355 g when the squat depth increases from 1 mm to 4 mm. The standard deviations for the 1 mm and 4 mm cases are 0.3434 g and 0.088 g, respectively. Similar results can be seen in Figure 11. The mean amplitude value increases from 0.3499 g to 1.6286 g when the squat depth increases from 1 mm to 4 mm. The standard deviations for the 1 mm and 4 mm cases are 0.1437 g and 0.7054 g, respectively. Squat F is located 12.18 m away from the point machine and squat G is 13.46 m away.

### 3.5. Linear Regression for Estimated Amplitude versus Squat Depth

One degree polynomial curve fitting was utilised to model the relationship between the peak-to-peak amplitude to the depth of squat G and F. This fitting used the least square error method. The dashed line in Figure 11 shows the fitted linear model for normalised peak-to-peak amplitude to the depth of squat F:

(4) y=0.255x+0.2942

where *y* is the normalised peak-to-peak amplitude value and *x* is the squat depth. The root mean squared error of the fit is 0.2578 g. The coefficient of determination $R^{2}$ is 0.7086, which means that the model predicts 70.86% of the variance in the variable *y*.

The dashed line in Figure 10 shows the fitted linear model for normalised peak-to-peak amplitude to the depth of squat G:

(5) y=0.388x+0.0452

where *y* is the normalised peak-to-peak amplitude value and *x* is the squat depth. The root mean squared error of the fit is 0.3231 g. The coefficient of determination $R^{2}$ is 0.7818, which means that the model predicts 78.18% of the variance in the variable *y*.

Unlike previous studies that focus on measuring squats on normal pieces of rails, this study showed that an alternative approach of installing accelerometers at the point machine is feasible, and the signals collected can be utilised to detect and estimate the squat defects of the S&C. Although these two linear models are preliminary and more data need to be collected in future experiments to validate them, those two models imply that increased squat depth should increase the normalised amplitude. These preliminary results did not consider a large variation of squat depth, which needs to be addressed in the future in the real environment.

## 4. Conclusions and Future Works

This study demonstrates it is possible to use the presented method to detect and quantify squats of different severities by processing the vibration signal measured at the point machine. In detail, the following conclusions are drawn:

- It is possible to extract and locate 6 out of 7 squats og 4 mm depth within a 13 m range from the accelerometer.
- It is possible to extract and locate 4 out of 6 squats of 1 mm depth that is within around a 13 m range from the accelerometer.
- It is challenging to extract and locate accurately both 1 mm and 4 mm depth squats that are further than around 22 m away from the accelerometer.
- The mean normalised amplitude value for squat F increases from 0.76 g to 1.24 g when the squat depth increases from 1 mm to 4 mm with standard deviations of 0.34 g and 0.09 g, respectively.
- The mean normalised amplitude value for squat G increases from 0.35 g to 1.63 g when the squat depth increases from 1 mm to 4 mm with standard deviations of 0.14 g and 0.71 g, respectively.
- It is possible to fit a linear model to the normalised amplitude versus squat depth for squats F and G with the data collected.

The conclusions above apply to the low-speed experiment under 2 m/s. The findings indicate that it is possible to compose an automatic squat detection algorithm with high accuracy if the squats are as deep as 4 mm and the squats are not more than around 13 m away. Since the experiment involves the bogie having a very low speed, the result might vary when the speed is much higher. However, it is reasonable that in a higher speed case, it would be easier to detect the squats on the rail with this approach, due to the increased energy of the impact force. The research method presented in this paper is still valid. This study is one of the first to propose such a new sensor installation approach. Although the data size is limited and some of the results need to be further verified, the possibility of proposing a new condition monitoring system for S&Cs was tested and analysed.

One possible future work could be to study the influence of curve, speed and weight of the wagon body and distances between the axles. The curve might not have an impact on the results, but a higher speed would probably result in higher amplitudes according to a previous study [24]. The difference of wagon body weights could likely be averaged out by collecting data from a large number of trains. The distance between the axles might be extracted from the vibration signal itself. Another possible future work could be to collect more data from a real railway system to verify the method presented in this study. Furthermore, yet another future work could be implementing a machine learning algorithm to learn from the patterns of the healthy S&Cs and perform continuous anomaly detection on them.

## Figures and Tables

**Table 1:** Squats’ dimension measurements.

General info			Size 1		Size 2	
**Rail Number**	**Squat Name**	**From** $S_{0}$	**Squat Diameter**	**Max Depth**	**Squat Diameter**	**Max Depth**
		**(m)**	**(mm)**	**(mm)**	**(mm)**	**(mm)**
4	A	5.7	43	1.2	62	3.7
4	B	6.7	41	1.0	61	3.9
1	C	7.27	42	1.0	63	3.7
3	D	10.68	42	1.0	66	4.4
1	E	12.47	0	0	65	3.7
3	F	18.04	42	1.1	65	4.2
1	G	19.32	42	1.0	64	3.7
1	H	28.02	42	1.5	62	4.7
3	I	29.23	42	1.4	62	4.3
3	J	32.14	42	1.2	63	4.4
1	K	34	42	1.1	61	4.1

**Table 2:** Parameters of the accelerometer used.

Name	Range (Hz)	Sensitivity (mV/g)	Destruction Limit (g)	Resonant Frequency (kHz)
KS91C	0.3−37,000	10 ± 20%	10,000	>60 (+25 dB)

**Table 3:** Test runs.

Test Scenario	Repetitions	Date
Without squats	3	31 March 2020
1 mm squats	3	6 April 2020
4 mm squats	3 (2 valid)	9 April 2020

**Table 4:** Squat detection for the 1 mm case.

Squat Name	Detected	Location Error < 0.5 m
A	Yes	Yes
B	Yes	Yes
C	No	No
D	No	No
E	N/A	N/A
F	Yes	Yes
G	Yes	Yes
H	No	No
I	No	No
J	No	No
K	No	No

**Table 5:** Squat detection for the 4 mm case.

Squat Name	Detected	Location Error < 0.5 m
A	Yes	Yes
B	Yes	Yes
C	Yes	Yes
D	No	No
E	Yes	Yes
F	Yes	Yes
G	Yes	Yes
H	No	No
I	No	No
J	No	No
K	No	No

## Data Availability

Not applicable.
